# Active vs. passive recovery during an aerobic interval training session in well-trained runners

**DOI:** 10.1007/s00421-022-04926-2

**Published:** 2022-03-09

**Authors:** Tania Sánchez-Otero, José Luis Tuimil, Daniel Boullosa, Adrián Varela-Sanz, Eliseo Iglesias-Soler

**Affiliations:** 1 Faculty of Sports Sciences and Physical Education, Department of Physical Education and Sport, University of A Coruna, Performance and Health Group, A Coruña, Spain; 2 Federal University of Mato Grosso do Sul, Campo Grande, Brazil; 3 College of Healthcare Sciences, James Cook University, Townsville, Australia; 4 Research and Development Department, iLOAD Solutions, Campo Grande, Brazil

**Keywords:** HIIT, Running, Red zone, Work interval, Rest interval

## Abstract

**Purpose:**

To compare cardio-metabolic, perceptual and neuromuscular responses to an aerobic interval training (AIT) running session, with active (AR) vs. passive recovery (PR).

**Methods:**

Eleven well-trained male distance runners (36.63 ± 6.93 years, 59.26 ± 5.27 mL·kg^−1^·min^−1^, ⁓ 35 min in 10 km) completed the University of Montréal Track Test (UMTT) and 2 AIT sessions on track in random order, which consisted of 4 × 2 min at 100% of the maximum aerobic speed (MAS), with 2 min of AR at 80% of the velocity associated to the second ventilatory threshold (vVT_2_), or no exercise (i.e., PR). During sessions, oxygen consumption (V̇O_2_), heart rate (HR), blood lactate [La], rating of perceived exertion (RPE), and countermovement jump (CMJ) were continuously monitored.

**Results:**

There were no differences in time spent in the “red zone” (i.e. > 90% V̇O_2max_) between sessions (222 ± 73 s AR vs. 230 ± 104 s PR, *p* = 0.588), although the PR exhibited a greater time spent at peak V̇O_2_ close to significance (117 ± 114 vs. 158 ± 109 s, *p* = 0.056). However, the AR elicited a higher mean V̇O_2_ (49.62 ± 5.91 vs. 47.46 ± 4.20 mL·kg^−1^·min^−1^, *p* = 0.021). The AR favored a lower [La] after sessions (6.93 ± 2.22 vs. 6.24 ± 1.93 mmol·L^−1^, *p* = 0.016) and a higher RPE during sessions (15 ± 0.45 vs. 14 ± 0.47, *p* = 0.045). Meanwhile, the CMJ was significantly potentiated during both sessions.

**Conclusion:**

Considering that PR elicited lower perceptual loading for a similar cardiorespiratory response, its use would be preferable, at least, for this type of AIT running sessions.

## Introduction

High-intensity interval training (HIIT) is a method that consists of performing repeated bouts (i.e., work intervals) above the anaerobic threshold, interspersed with pauses, thus allowing the accumulation of more time at a targeted intensity during a single training session (Billat et al. 2000; Tuimil et al. 2011; Buchheit and Laursen 2013). Following Buchheit and Laursen (2013), HIIT protocols are often performed at intensities close to the maximum oxygen consumption (V̇O_2max_), with shorter bouts (≤ 60 s) typically performed above V̇O_2max_, and longer bouts (> 60 s) performed at or below V̇O_2max_. Based on the intensity of bouts, HIIT can be performed taxing more the anaerobic (e.g., sprint interval training, SIT) or the aerobic (i.e., aerobic interval training, AIT) metabolism (Buchheit and Laursen 2013; Schoenmakers et al. 2019). In this regard, Hill and Rowell (1997) had previously suggested in the late 90s that bout duration should be, at least, > 60% of the time limit (T_lim_) at the velocity associated to V̇O_2max_ or maximum aerobic speed (MAS) for reaching V̇O_2max_ in AIT running sessions. Thus, prescription of HIIT sessions includes the manipulation of intensity and duration of bouts and recovery intervals for designing appropriate training workloads when looking for specific metabolic and neuromuscular adaptations in the short and the long term (Buchheit and Laursen 2013).

Pause duration is determined by the duration of the bout and its intensity, to allow a proper recovery for successfully performing the subsequent work interval, and it is based on the work-to-rest ratio or the return of some physiological parameters (e.g., heart rate – HR, muscle oxygen consumption) to a set value (Foster et al. 2017; Schoenmakers et al. 2019; Schoenmakers and Reed 2019; Fennell and Hopker 2021b). While duration of pauses appears not to be important for the total physiological strain of a HIIT session unless the recovery duration was very short (Smilios et al. 2018; Schoenmakers et al. 2019), the intensity of pauses, with selection of active recovery (AR) vs. passive recovery (PR), is an issue which is still under debate. In this regard, Buchheit and Laursen (2013) suggested that AR could be recommended for longer recovery intervals of 3–4 min, while PR could be more beneficial for shorter recovery intervals of < 3 min. These recommendations were based not only on the plausible positive effect of AR on oxygen kinetics, and therefore on the total time spent in the so-called “red zone”, which implies to attain an exercise intensity of at least 90% of V̇O_2max_ (Midgley et al. 2006; Buchheit and Laursen 2013), but also on its negative effect on performance capacity as AR can lower muscle oxygenation (Dupont et al. 2007; Buchheit et al. 2009) and phosphocreatine re-synthesis (Spencer et al. 2006) when interval recovery time is < 3 min. In fact, recent scientific evidence demonstrates that longer AR durations (i.e., 4 min) reduce blood lactate concentration significantly more than shorter ones (i.e., 2 min), suggesting that a short AR may activate anaerobic glycolysis to a greater extent in the subsequent work intervals as a result of accumulated fatigue (Smilios et al. 2018). Paradoxically, these previous recommendations were mostly based on studies performing SIT (Dupont et al. 2007; Buchheit et al. 2009) and not typical AIT sessions. Thus, several studies have investigated the effects of AR and PR on running performance (Tardieu-Berger et al. 2004; Abderrahman et al. 2013), accumulated time in the “red zone” (i.e. > 90% V̇O_2max_) (Thevenet et al. 2007a, b) and on metabolic and hormonal parameters (e.g., plasma glucose and gluco-regulatory hormones, such as adrenaline and noradrenaline) (Abderrahmane et al. 2013; Abderrahman et al. 2018), during SIT-based running sessions (e.g., 2 × 8–15 × 30 s at 100–110% of MAS, interspersed with 30 s AR at 50% of MAS or PR). Taking together, these previous studies suggest that time spent in the “red zone” did not significantly differ between recovery modes, whereas PR may lead to greater running performance (i.e., longer T_lim_ or more repetitions performed) and AR can improve some metabolic and hormonal parameters.

More recently, it has been suggested that PR leads to a greater performance during HIIT sessions in both SIT and AIT modalities (Perrier-Melo et al. 2020). Specifically, a recent study with cyclists showed that PR during AIT sessions may facilitate a greater external training load for similar physiological responses while lowering perceived exertion (Fennell and Hopker 2021a). Further, previous evidence has highlighted the important role that perception of effort plays during high-intensity aerobic exercise for exercise tolerance among highly motivated individuals (Marcora et al. 2010). Additionally, AR after self-paced long bouts of cycling exercise (e.g., 4 min or 8 min) may elicit longer time accumulated in the “red zone”, especially when the work-to-rest ratio is 2:1 (Dall’ Agnol et al. 2021).

On the other hand, previous studies have demonstrated the appropriateness of simple explosive exercises involving the stretch–shortening cycle as jumps (i.e., countermovement jump, CMJ) for assessing lower limbs neuromuscular function in endurance runners of different levels (Boullosa and Tuimil 2009; Boullosa et al. 2011, 2018; García-Pinillos et al. 2015, 2021). In this regard, it has been also suggested that a better potentiation/fatigue balance would be expected in endurance-trained athletes during and after conditioning activities as is the case of interval training running sessions (Boullosa et al. 2018). However, to the best of our knowledge, there is no evidence on the role that intensity during recovery might play for perceptual, physiological and neuromuscular responses during AIT running sessions, particularly when performed in the field by well-trained endurance runners. Previous studies focused on the effects of SIT sessions with different populations rather than endurance runners. This knowledge is important from a practical point of view to better select the mode of recovery depending on the session objectives.

Therefore, the aim of this investigation was to compare the use of AR vs. PR during a typical AIT running session (4 × 2 min at 100% of MAS, with a work-to-rest ratio of 1:1) performed on a 400-m outdoor track on metabolic (i.e., cardiorespiratory and blood lactate [La]), neuromuscular (i.e., jump capacity) and perceptual (i.e., rating of perceived exertion, RPE) responses in well-trained endurance runners. Based on previous literature (Fennell and Hopker 2021a), our hypothesis was that PR would favor a lower internal load (i.e., physiological and neuromuscular effects: V̇O_2_, HR, [La], RPE, jump height/jump power) for a similar external training load (i.e., volume × intensity: total covered running distance, running speed).

## Methods

### Participants

Eleven well-trained male runners who frequently competed at regional and national level in running races (10 km personal best 2101 ± 144 s) volunteered for participation. The main characteristics of the participants are shown in Table 1. Participants read and signed an informed consent form before the evaluations and they could withdraw from the study at any time. The study was approved by the University of A Coruna Ethics Committee and was conducted according to the tenets of the Declaration of Helsinki.

**Table 1:** Characteristics of the participants

	Mean ± SD
Age (years)	36.63 ± 6.93
Height (cm)	174.78 ± 7.16
Body mass (kg)	71.31 ± 10.31
BMI (kg·m^−2^)	23.21 ± 1.77
10 km best (s)	2101 ± 144
MAS (km·h^−1^)	18.72 ± 1.05
T_UMTT_ (s)	1407.27 ± 126.89
VO_2max_ (mL·kg^−1^·min^−1^)	59.26 ± 5.27
HR_max_ (bpm)	178.09 ± 7.40
vVT_2_ (km·h^−1^)	15.45 ± 0.93
HR_VT2_ (bpm)	160.57 ± 6.12
T_lim_ (s)	177.33 ± 37.96

*BMI* body mass index; 10 km best 10 km personal best; *MAS* maximum aerobic speed; *TUMTT* total completed time in the University of Montréal Track Test; *VO*_*2max*_ maximum oxygen uptake attained during the University of Montréal Track Test; *HR*_*max*_ maximum heart rate; *vVT2* velocity associated to the second ventilatory threshold; *HR*_*VT2*_ heart rate associated to the second ventilatory threshold; *T*_*lim*_ time limit at 105% of MAS in the square-wave supramaximal running test during the verification phase; *SD* standard deviation

### Design and methodology

**Fig. 1:**
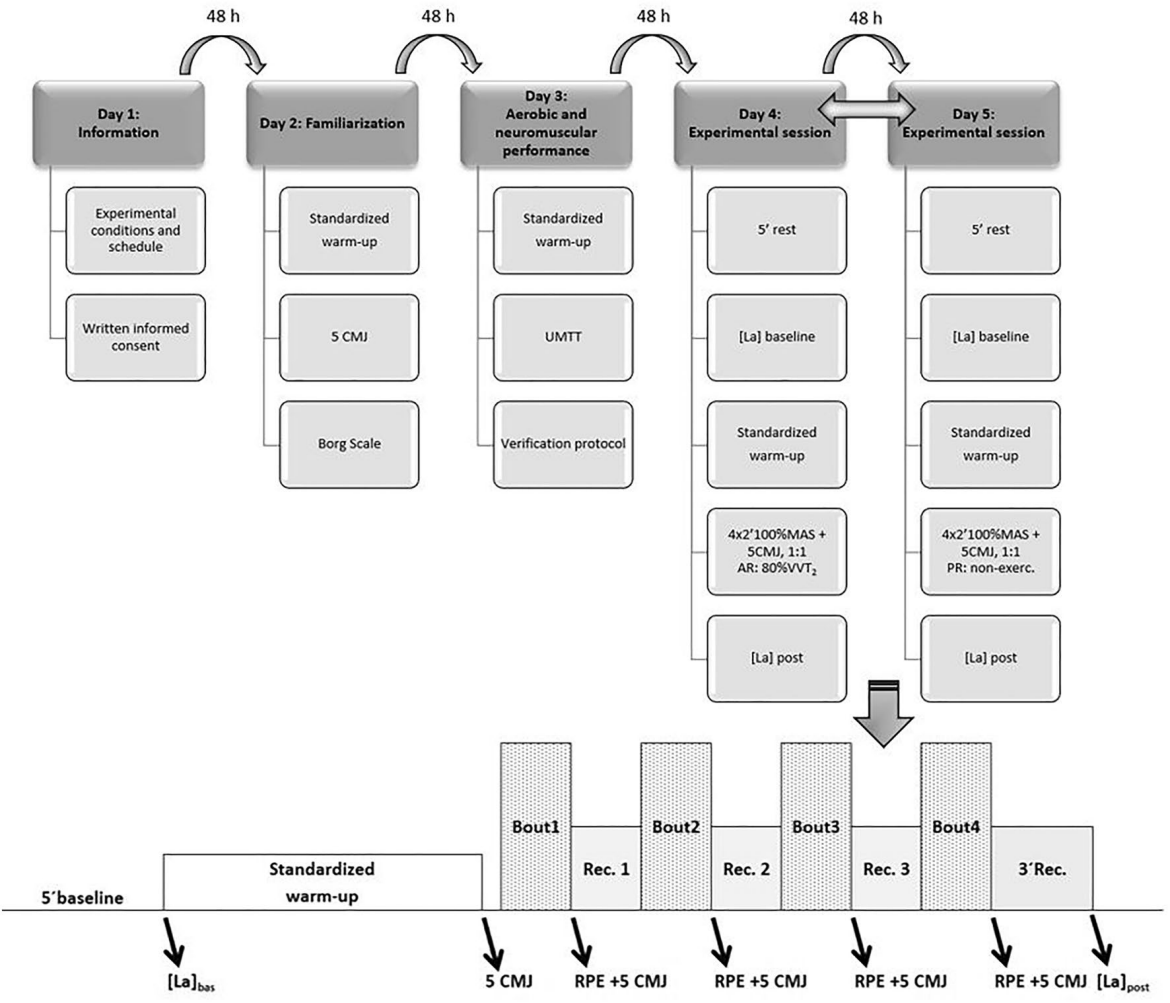
Experimental design schedule and aerobic interval training protocol scheme. *HR* heart rate; *CMJ* countermovement jump; *UMTT* University of Montréal Track Test; *MAS* maximum aerobic speed; 1:1 work-to-rest ratio in the aerobic interval training session; *AR* active recovery; *vVT2* velocity associated to the second ventilatory threshold; *PR* passive recovery; *Bout* work interval; *Rec*. recovery; *[La]* blood lactate concentration; *RPE* rating of perceived exertion. *Note* the two-headed arrow between experimental sessions on day 4 and day 5 means that these sessions were performed in a randomized order. All sessions were separated by, at least, 48 h

Our investigation was a cross-sectional study performed along 5 testing sessions separated by a minimum of 48 h (see Fig. 1).

#### Day 1: Information session

The first session was devoted to explain the study procedures and to instruct the participants about experimental conditions, which implied to avoid the ingestion of food, alcohol, caffeine and/or tobacco during the 3 h before evaluations, and not to perform any high-intensity effort during the 24 h prior to each session. Participants were also instructed not to modify their nutrition patterns during the study.

#### Day 2: Familiarization session

The second session consisted of a familiarization with testing procedures. After a standardized warm-up, participants performed 5 continuous CMJs on a force plate (Quattro-Jump, Kistler Instrument, Switzerland) and were also instructed about the Borg’s 6–20 RPE scale.

#### Day 3: Determination of aerobic and neuromuscular performance

In the third session, cardiorespiratory parameters during an incremental test on track until exhaustion were obtained. First, participants completed a standardized warm-up consisting of 10 min of running at 60% of the estimated maximum HR (HR_max_) calculated according to Tanaka et al. (2001), 5 min of calisthenics, 5 sets of 50 m at a progressive velocity, 5 CMJs on the force plate, and 5 min of rest. After that, the gas analyzer (K4b2, Cosmed, Italy) was calibrated according to manufacturer instructions prior to perform the University of Montréal Track Test (UMTT). The UMTT was carried out on a 400-m outdoor track following the original protocol (Léger and Boucher 1980) (i.e., 1 km·h^−1^ every 2 min, starting at 8 km·h^−1^) but with a cyclist setting the running pace (Boullosa and Tuimil 2009). Participants were verbally encouraged to run until volitional exhaustion. The RPE scale was fixed on the back of the cyclist to record the RPE at the end of each stage. Immediately after finishing the UMTT, a blood sample was obtained from the earlobe to determine [La] after the test using a portable blood lactate analyzer (Lactate Scout, SensLab GmbH, Germany).

The MAS was considered as the speed attained in the final completed stage, and calculated more precisely according to Kuipers et al. (1985). The total time at the end of the UMTT (T_UMTT_) was also recorded.

Breath-by-breath raw V̇O_2_ and HR data were automatically filtered with a custom-made software and subsequently averaged to 15-s intervals. The V̇O_2max_ and HR_max_ were considered as the highest V̇O_2_ and HR attained in 2 successive 15-s periods, respectively. To confirm a maximal effort, at least 2 of the following criteria were observed in all participants: a) a plateau of V̇O_2_ despite increasing the running speed (change in V̇O_2_ ≤ 150 mL·min^−1^); b) maximum respiratory exchange ratio ≥ 1.1; c) HR_max_ ≥ 95% age-predicted maximum HR; d) maximum [La] ≥ 8 mmol·L^−1^; and e) RPE ≥ 17 (Midgley et al. 2007).

The first and the second ventilatory thresholds were visually determined by two researchers following traditional criteria (Binder et al. 2008).

After exhaustion in the UMTT, participants rested by walking or standing for 15 min. Subsequently, they performed a square-wave supramaximal running test (i.e., T_lim_) at 105% of MAS. This verification procedure has been previously described (Sánchez-Otero et al. 2014).

#### Days 4 and 5: experimental sessions

The experimental sessions (i.e., AR and PR sessions) were carried out in the fourth and fifth sessions under thermo-neutral environmental conditions for all participants (i.e. < 24 °C and < 80% of relative air humidity). The participants performed a standardized warm-up at the beginning of each session. The sessions consisted of 4 bouts of 2 min at 100% of MAS, with a work-to-rest ratio of 1:1. In one session, the recovery period between bouts was passive (i.e., no exercise), whereas in the other session, the rest interval was active (i.e., running at 80% of the velocity associated to the second ventilatory threshold, vVT_2_). Participants ran on a 400-m outdoor track following a cyclist who set the running pace, and were instructed to run 2–3 m behind the cyclist and to maintain an even pace for homogenizing drafting between experimental conditions (Zouhal et al. 2015). The order of both sessions was randomized and they were separated by, at least, 48 h.

Blood lactate concentration was recorded at rest before each session and 3 min after finishing the last bout. The CMJ protocol was performed immediately after each bout, and RPE was also recorded. Maximum jump height, mean jump height, peak power and vertical stiffness (*kvert*) of 5 jumps were recorded for further analysis. *Kvert* was calculated as the quotient of the change in ground reaction force and displacement of the center of mass (Mudie et al. 2017), normalized by body mass (Boullosa et al. 2011).

Cardiorespiratory parameters were obtained after the proper calibration of the gas analyzer, which was fixed to the athlete. Breath-by-breath raw VO_2_ and HR data were automatically filtered with a custom-made software and subsequently averaged to 5-s intervals. Only cardiorespiratory data obtained during bouts and recovery intervals were analyzed for both AR and PR sessions (cardiorespiratory data obtained during the CMJ protocol, immediately performed after each bout, were eliminated). Participants did not attain their V̇O_2max_ nor their HR_max_ during the experimental sessions, thus peak V̇O_2_, mean V̇O_2_, peak HR and mean HR were recorded. The time-to-reach 90% of V̇O_2max_ (TTR90% V̇O_2max_) and peak V̇O_2_ (TTRpeak V̇O_2_), and the accumulated time at or over 90% of V̇O_2max_ (TT90% V̇O_2max_) and at peak V̇O_2_ (TTpeak V̇O_2_ were also analyzed. A schematic representation of the protocol is shown in Fig. 1.

### Statistical analysis

Data are reported as mean ± SD and ranges. The normality assumption for each parameter was verified with the Shapiro–Wilk test. For comparisons between sessions, a paired *t* tests or a Wilcoxon signed-rank test was performed when appropriate. A two-way repeated measures of analysis of variance (ANOVA) was performed with session and bout as factors. When a significant session × bout interaction was detected, a post hoc *t* test was carried out with Bonferroni’s adjustment. If normality was rejected, a non-parametric ANOVA type test (Noguchi et al. 2012) was employed, performing post hoc comparisons with a Wilcoxon signed-rank test with Bonferroni’s correction. Effect sizes for parametric ANOVA are reported as partial eta squared (pη^2^), whereas for significant pairwise contrasts are presented as Hedge’s g (G) and matched-pairs rank-biserial correlation (*r*) for parametric and non-parametric comparisons, respectively. The statistical power for the interaction of the 2 × 4 repeated measures ANOVA with a sample size of 11, a correlation among repeated measures of 0.7 and a medium effect size (*f* = 0.30) is 0.83. Additionally, we calculated the sensitivity of the repeated measures ANOVA to detect this interaction for an alpha level of 0.05, a power of 0.80, a total sample of 11 subjects, and a correlation between repeated measurements of 0.7, obtaining that the test was sensitive to detect a medium effect size (*f* = 0.29). Main analyses were carried out using the statistical package SPSS version 20.0 (SPSS, IBM, Armonk, NY, USA), while non-parametric ANOVA type analysis and rank-biserial correlation were performed using the nparLD R and rcompanion software package for R (R software v3.6.1. R Foundation, Vienna, Austria), respectively. The level of statistical significance was set at 0.05.

## Results

**Fig. 2:**
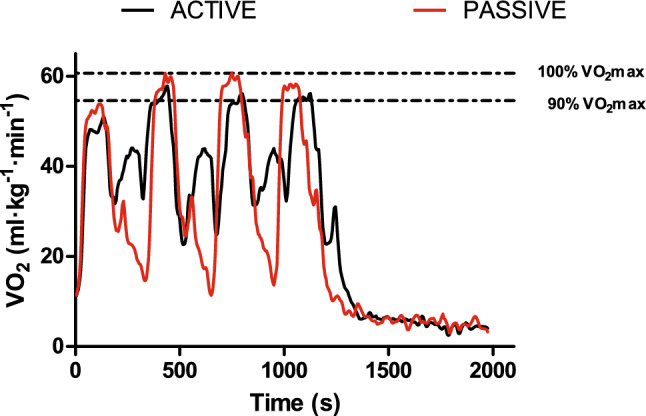
Oxygen kinetics from the same runner during bouts and recovery intervals for both AR and PR sessions. Black line represents VO_2_ kinetics during the AR session, whereas red line represents VO_2_ kinetics during the PR session. Dotted horizontal line delimits the so-called “red zone” (i.e., intensity between 90 and 100% VO_2max_)

Table 2 shows VO_2_ demands, both in absolute and relative (% V̇O_2max_) values, total covered distance, time spent in the “red zone”, and time-to-reach the “red zone” during bouts in AR and PR sessions. Higher mean V̇O_2_ values (*r* = 0.79; 95% CI [0.333,1]) and mean V̇O_2_ relative to V̇O_2max_ (*G* = 0.479; 95% CI [0.142,0.817]) were recorded during the session with AR, whereas higher peak V̇O_2_ values relative to V̇O_2max_ were obtained during the PR session (*G* = 0.345; 95% CI [0.039,0.655]). Oxygen kinetics during bouts and recovery intervals from the same runner during both sessions is shown in Fig. 2.

**Table 2:** VO_2_ demands, total covered distance, time-to-reach the “red zone” and time spent in the “red zone” during bouts in both AR and PR sessions

	AR	PR	*p* value
	Mean ± SD	Mean ± SD	
Mean VO_2_ (mL·kg^−1^·min^−1^)	49.62 ± 5.10	47.46 ± 4.20	0.021^w^
Mean VO_2_ (% VO_2max_)	83.82 ± 5.96	80.59 ± 6.49	0.014
Peak VO_2_ (mL·kg^−1^·min^−1^)	56.43 ± 5.91	57.86 ± 5.85	0.131^w^
Peak VO_2_ (% VO_2max_)	95.11 ± 5.81	97.85 ± 7.55	0.047
Distance (m)	3733.33 ± 210.65	2496.97 ± 140.99	< 0.001
TTpeakVO_2_ (s)	117 ± 114	158 ± 109	0.056
TT90%VO_2max_ (s)	222 ± 73	230 ± 104	0.588
TTRpeakVO_2_ (s)	61 ± 24	58 ± 9	0.906
TTR90%VO_2max_ (s)	46 ± 11	45 ± 7	0.735

*AR* active recovery session; *PR* passive recovery session; *VO*_*2*_ oxygen uptake; Distance total covered distance in the experimental sessions*; TTpeakVO*_*2*_ total time at peak VO_2_; *TT90%VO*_*2max*_ total time at or over 90%VO_2max_; *TTRpeakVO*_*2*_ time-to-reach peak VO_2_; *TTR90%VO*_*2max*_ time-to-reach the 90% of VO_2max_; *SD* standard deviation. w next to a *p* value indicates that this corresponds to a Wilcoxon signed-rank test

*Note*: For a better reader’s comprehension, VO2max registered in the UMTT was not attained during AR and PR sessions, thus absolute peak VO2 and relative to VO2max values are presented

**Fig. 3:**
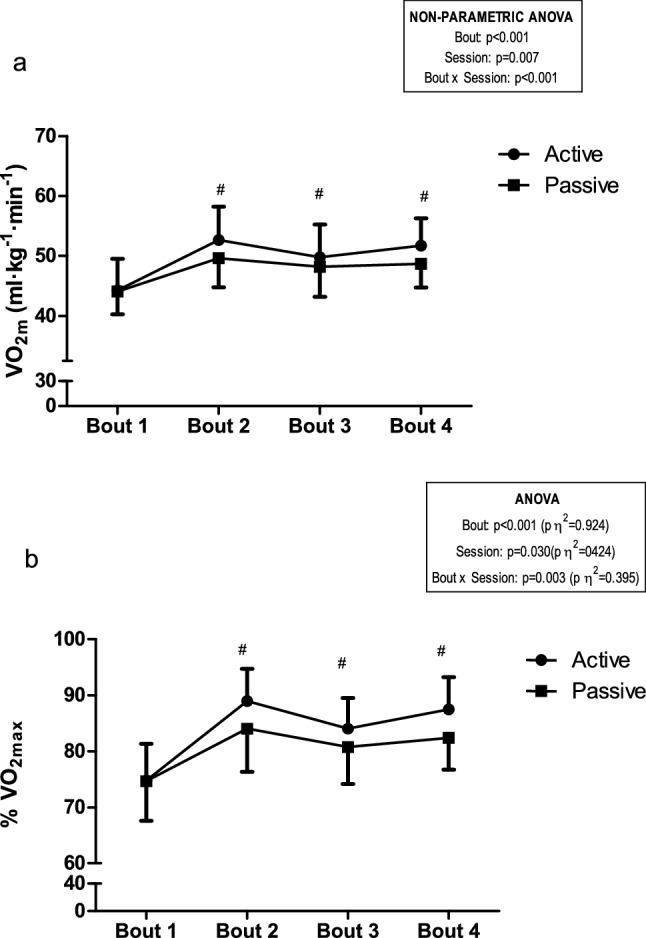
Mean oxygen uptake **a** and mean oxygen uptake expressed as a percentage of the maximum oxygen uptake **b** throughout bouts for both AR and PR sessions. ^#^Significant differences between experimental sessions. *Note*: For a better reader’s comprehension and relevance, only significant differences between experimental sessions are shown

Absolute mean V̇O_2_ and mean V̇O_2_ expressed as a percentage of V̇O_2max_ throughout the bouts are represented in Fig. 3.

Recorded HR values during running bouts were significantly higher during AR when compared to PR for mean HR (158.56 ± 7.92 vs 151.81 ± 5.48 bpm, *p* = 0.001; G = 0.853, 95% CI [0.348, 1.358]) and peak HR (169.28 ± 3.63 vs 166.34 ± 4.59 bpm, *p* = 0.05; G = 0.636, 95% CI [0.166, 1.106]). A significant effect of bout (*p* < 0.001; *pη*^2^ = 0.993), session (*p* = 0.003; *pη*^2^ = 0.648) and session × bout interaction (*p* = 0.01; *pη*^2^ = 0.510) were found for mean HR throughout bouts. Post hoc comparisons detected higher values in PR in comparison with AR for bout 2 (*p* = 0.001; G = 1.122, 95% CI [0.565, 1.680]), bout 3 (*p* = 0.002; *G* = 0.842, 95% CI [0.339, 1.344]) and bout 4 (*p* < 0.001; *G* = 1.134, 95% CI [0.574, 1.694]). Similarly, mean HR during recovery intervals was significantly higher during AR when compared to the PR session (142.64 ± 8.49 vs 114.64 ± 7.53 bpm, *p* = 0.001; *G* = 3.194, 95% CI [2.077, 4.311]).

A significant effect of bout (*p* < 0.001; *pη*^2^ = 0.883) and session (*p* = 0.016; *pη*^2^ = 0.454), with no session × bout interaction, was found for [La] recorded 3 min after finishing the last bout during the sessions, with higher values for PR when compared to AR session (6.93 ± 2.22 vs 6.24 ± 1.93 mmol·L^−1^, respectively).

Table 3 shows the changes for CMJ parameters during sessions. The ANOVA did not detect any significant session × bout interaction for maximum jump height (*p* = 0.199), mean jump height (*p* = 0.729), peak power (*p* = 0.245), and kvert (*p* = 0.289). A main effect of bout was only detected for mean jump height (*p* = 0.004; *pη*^2^ = 0.443) and peak power (*p* < 0.001; *pη*^2^ = 0.518).

**Table 3:** Changes in CMJ parameters throughout the session with AR and PR

	AR	PR
	Mean ± SD	Mean ± SD
H_mpre-bout_ (cm)	23.32 ± 3.99	23.98 ± 2.93
H_m_.B1 (cm)*^#^	23.62 ± 2.96	24.35 ± 2.62
H_m_.B2 (cm)	24.67 ± 3.39	24.81 ± 3.29
H_m_.B3 (cm)	24.59 ± 3.23	24.79 ± 2.76
H_m_.B4 (cm)^ɸ^	25.06 ± 3.24	25.45 ± 3.22
H_maxpre-bout_ (cm)	25.37 ± 5.06	25.71 ± 3.30
H_max_.B1 (cm)	26.95 ± 4.66	26.28 ± 3.07
H_max_.B2 (cm)	27.95 ± 5.03	26.03 ± 3.67
H_max_.B3 (cm)	25.69 ± 3.44	27.31 ± 3.81
H_max_.B4 (cm)	27.20 ± 4.37	27.42 ± 3.27
P_peakpre-bout_ (W·kg^−1^)	39.13 ± 5.62	39.63 ± 5.21
P_peak_.B1 (W·kg^−1^)	40.25 ± 4.90	40.29 ± 3.61
P_peak_.B2 (W·kg^−1^)^ɸ^	42.04 ± 5.26	40 ± 6.44
P_peak_.B3 (W·kg^−1^)^ɸ^	41.55 ± 5.16	40.83 ± 3.78
P_peak_.B4 (W·kg^−1^)^ɸ^	42.02 ± 5.32	40.83 ± 4.73
*kvert*_pre-bout_ (N·m^−1^·kg^−1^)	68.02 ± 17.65)	71.73 ± 22.79
*kvert*.B1 (N·m^−1^·kg^−1^)	73.71 ± 14.82	73.5 ± 15.49
*kvert*.B2 (N·m^−1^·kg^−1^)	77.11 ± 16.32	73.82 ± 17.15
*kvert*.B3 (N·m^−1^·kg^−1^)	73.01 ± 11.74	72.05 ± 16.64
*kvert*.B4 (N·m^−1^·kg^−1^)	72.68 ± 13.21	71.46 ± 14.80

*Note:* The different CMJ variables values with “pre-bout” sub-index refer to the 5 CMJ performed prior to the first AIT work interval

*AR* active recovery session; *PR* passive recovery session; *H*_*m*_ mean height reached during the jump; *H*_*max*_ maximum height reached during the jump; *P*_*peak*_ peak power; *kvert* leg stiffness; *B1* bout 1; *B2* bout 2; *B3* bout 3; *B4* bout 4; *SD* standard deviation

Post hoc contrasts for significant main effects of bout factor detected by ANOVA:

*Significantly different from B2 (*p* < 0.05)

^#^Significantly different from B4 (*p* < 0.05)

^ɸ^Significantly different from pre-bout (*p* < 0.05)

**Fig. 4:**
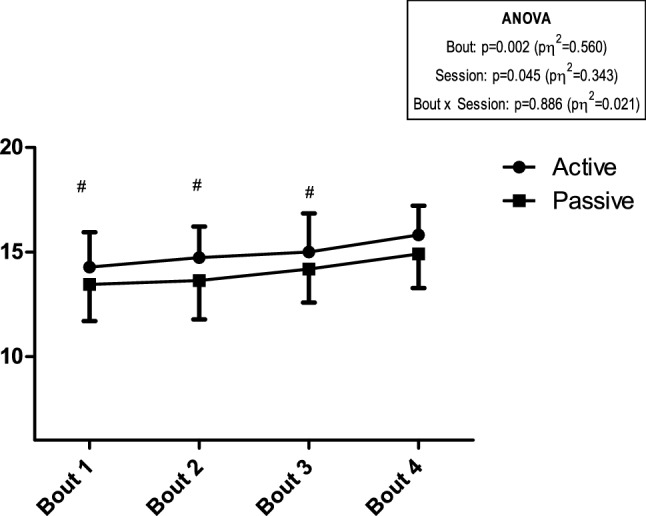
Rating of perceived exertion recorded throughout AR and PR sessions. ^#^Significantly lower in comparison with Bout 4 (*p* < 0.05) for post-hoc contrasts regarding main effect of bout

Finally, descriptive and ANOVA results regarding RPE are presented in Fig. 4. A significant effect of bout (*p* = 0.002; *pη*^2^ = 0.560) and session (*p* = 0.045; *pη*^2^ = 0.343), with no session × bout interaction, was revealed with AR showing higher RPE values when compared to PR session (14.95 ± 1.66 vs. 14.05 ± 1.75, respectively).

## Discussion

To the best of our knowledge, this is the first study simultaneously analyzing cardiorespiratory, metabolic, neuromuscular, and perceptual responses during an AIT running session with AR vs. PR intervals. The main findings of the current study are: (1) as expected, the AR elicited higher mean V̇O_2_, mean HR and peak HR levels during running bouts; (2) the peak V̇O_2_ relative to V̇O_2max_ during bouts was higher with PR; (3) the mean V̇O_2_ and HR values during recovery intervals were lower with PR; 3) the peak lactate concentration was globally lower with AR; (4) the jump capacity was potentiated, independently from recovery modes; (5) the perceived effort was higher with AR; 6) total covered distance was greater with AR.

The finding of greater mean V̇O_2_ values in the session with AR is not surprising and agrees with previous literature. However, it should be noted that most previous studies used shorter supramaximal bouts (i.e., SIT) and not all of them were performed with runners. This is an important consideration as the training session selected, in terms of intensity and volume, could be considered more appropriate for the level of the recreational runners of our study (i.e., 10 km personal best of ⁓35 min). In this regard, O’Brien et al. (2008) demonstrated that a typical AIT running session consisting of 5 bouts of 2 min at MAS with 2 min AR at 50% of MAS leads to a higher mean V̇O_2_ (3200 ± 661 mL/min) and more time spent in the “red zone” (4.47 ± 3.55 min) during the session than 1-min bouts (3076 ± 604 mL/min and 1.32 ± 3.94 min, respectively) or continuous running (2909 ± 584 mL/min) of equivalent external load. On the other hand, Seiler and Sjursen (2004) reported similar mean V̇O_2_ values relative to V̇O_2max_ (⁓61% VO_2max_) when performing 12 self-paced bouts of 2 min interspersed with 2-min self-paced recovery intervals compared to 1-min bouts in a sample of well-trained runners. The higher mean V̇O_2_ values attained during bouts (ranging from 81 to 84% V̇O_2max_ in both conditions) could be explained by the differences in volume and intensity of our protocol in comparison to that used by Seiler and Sjursen (2004) (i.e., more bouts and self-paced). Moreover, peak V̇O_2_ values relative to V̇O_2max_ reported by Seiler and Sjursen (2004) (92% V̇O_2max_) were similar to those obtained in our study (ranging from 95 to 98% VO_2max_ in both conditions).

Taking together, this idea supports the previous recommendations of Hill and Rowell (1997) of using longer bouts during AIT sessions for maximizing the time spent in the “red zone” and therefore, improving endurance performance (Hill and Rowell 1997; Buchheit and Laursen 2013). Further, this would be reinforced in our study considering that the duration of prescribed bouts for both AR and PR sessions lasted longer (i.e., 120 s) than 60% of the T_lim_ at 105% of MAS (⁓106 s) during the verification phase. In this sense, we found non-significant differences between experimental sessions regarding TT90% V̇O_2max_, TTpeak V̇O_2_, TTR90% V̇O_2max_ and TTRpeak V̇O_2_. However, and although these variables were quite similar for both conditions, we found longer TTpeak V̇O_2_ in the PR session with confidence interval of the effect size not including null effect (*p* = 0.056; *G* = 0.341, 95% CI [0.022,0.660]). This phenomenon could be explained by a more pronounced increase in V̇O_2_ rate for the PR condition due to a greater V̇O_2_ amplitude (i.e., the difference regarding V̇O_2_ demands between the start of a bout and the attainment of the “red zone”), resulting in a steeper V̇O_2_ slope (i.e., better V̇O_2_ kinetics; see Fig. 2 as an example) since the time-to-reach the “red zone” was similar for both conditions. Our results and rationale are in accordance with Fennell and Hopker (2021a), who also found a non-significant difference in time spent in the “red zone” between conditions in a recent investigation performed with well-trained cyclists. These authors argued that PR may reduce V̇O_2_ demands at the start of the subsequent bout during AIT sessions, eliciting a higher V̇O_2_ amplitude and reducing the time-to-reach not only a V̇O_2_ plateau but also the “red zone”, thus suggesting better V̇O_2_ kinetics (Fennell and Hopker 2021a). Further, it was previously demonstrated that PR facilitates a greater interval performance (e.g., capability of performing more bouts or maintenance of a higher speed or power in subsequent bouts) with a similar physiological stress when compared to AR in both SIT-based (Tardieu-Berger et al. 2004; Thevenet et al. 2007b; Abderrahman et al. 2013; Perrier-Melo et al. 2020) and AIT-based (Perrier-Melo et al. 2020; Fennell and Hopker 2021a) HIIT sessions. The fact that our runners completed only 4 bouts of 2 min at 100% of MAS, experiencing a significantly lower mean V̇O_2_, mean HR, peak HR and RPE for a similar external load with PR, may suggest that they could be able to perform more bouts in this condition and, therefore, accumulate more time in the “red zone”. In this regard, it is also worthy to mention that total covered distance was significantly greater during the session with AR (i.e., ⁓ 1.2 km). However, this increment in total running distance should be cautiously interpreted, since it was performed at low intensities (i.e., 80% of vVT_2_ during recovery intervals). In this sense, one must consider that total external training load (i.e., including bouts and rest intervals) will be greater for AR when compared to PR sessions. However, targeted training intensities during work intervals were identical for both conditions. Thus, from a practical point of view, the distance accumulated during AIT sessions with AR should be considered in the computed training volume. From the current results, it can be suggested the use of PR when targeting for accumulating a greater volume of high-intensity work intervals, whereas AR could be recommended for maximizing physiological stress during AIT running sessions of moderate volume. This suggestion is based on the lower mean HR and V̇O_2_ values recorded during the PR intervals when compared to the active ones. Meanwhile, the correspondence between V̇O_2_ and HR responses during running bouts does confirm the validity of HR as a simple and valid monitoring tool to verify the purported physiological adaptations during HIIT sessions.

On the other hand, peak lactate after the experimental sessions was significantly lower when AR was performed at 80% of vVT_2_. These results are in accordance with previous studies which also demonstrated a better lactate clearance during AIT running sessions when active recovering at velocities close to the anaerobic threshold (Menzies et al. 2010).

One interesting finding was the post-activation performance enhancement evidenced by higher mean jump height and peak power values recorded at the end of the last bouts in both conditions. This finding is in agreement with previous reports with endurance runners of different levels and sex after different running exercises. Previously, García-Pinillos et al. (2015) observed that some runners (i.e., responders) exhibited jump potentiation but others did not (i.e., non-responders) during a 4 × 3 × 400 m intervals with 1 min of PR between bouts and 3 min between sets. While differences between sessions and used methods make comparisons difficult, our results show that the expected greater neuromuscular fatigue associated to AR was not observed as both sessions elicited similar responses. Further, the jumping height potentiation was accompanied by a preserved vertical stiffness which confirm a reduced neuromuscular fatigue during the sessions. This may suggest that the completion of additional bouts by our subjects would be feasible. Meanwhile, the regular use of vertical jump evaluations during HIIT sessions can be recommended to monitor the neuromuscular impact of different HIIT sessions to simultaneously evaluate the acute and chronic effects of different HIIT schemes (Boullosa et al. 2018; García-Pinillos et al. 2021).

Finally, we observed lower RPE scores for the PR condition at different time points. Moreover, recorded RPE scores constantly increased during bouts for both conditions. This is in accordance with previous studies since it was suggested that perception of effort increase linearly during AIT sessions and will be felt “hard” (i.e., RPE 15–16) initially and perceived as “very hard” (i.e., RPE 17–18) by the end of the workout (Seiler and Sjursen 2004). In addition, considering the lower RPE values throughout the session with PR, it would be hypothesized that runners in this condition would be able to complete more bouts until exhaustion, as perception of effort plays an important role in exercise tolerance during high-intensity aerobic exercise (Marcora et al. 2010). Further studies should be conducted to elucidate if other recovery work-to-rest ratios would influence the perceptual responses that can be easily recorded by practitioners during HIIT sessions. In this regard, it would be interesting to see how these RPE values would result in different session RPE (sRPE) values.

The present study has some limitations. Although participants were instructed not to change their habitually nutrition patterns, we did not control the diet during the course of the investigation. Another point to mention is the heterogeneity level presented by our runners, since 10 km personal best ranged from ⁓ 32 min to 38 min 30 s. The extrapolation of our results might not be appropriate for athletes with different characteristics (e.g., level, sex, etc.) and other AIT running sessions with different schemes and work-to-rest ratios.

## Conclusion

For the first time, we have concurrently analyzed the cardio-metabolic, neuromuscular and perceptual response of AIT running sessions completed on a 400-m outdoor track at 100% of MAS but differing in recovery mode. The practical applications from the current results for coaches, sport scientist and athletes are different regarding training objectives. Recreational runners as those of our study could benefit from AR during AIT running sessions when looking for a higher physiological stress. In contrast, PR may elicit a lower physiological stress for a similar external training load and, therefore, could be preferable for high-volume AIT running sessions. However, it is still to be solved if the same picture would be evident with a longer session with more running bouts. Further, longitudinal studies are guaranteed to better understand the adaptations in the long-term to these recovery modes during AIT running sessions.

## Data Availability

Not applicable.

## Abbreviations

AIT: Aerobic interval training
ANOVA: Analysis of variance
AR: Active recovery
CMJ: Countermovement jump
HIIT: High-intensity interval training
HR: Heart rate
HR_max_: Maximum heart rate
[La]: Blood lactate concentration
MAS: Maximum aerobic speed
PR: Passive recovery
RPE: Rating of perceived exertion
SIT: Sprint interval training
T_lim_: Time limit
TT90%V̇O_2max_: Accumulated time at or over 90% of V̇O_2max_
TTR90%V̇O_2max_: Time-to-reach 90% of V̇O_2max_
TTRpeakV̇O_2_: Time-to-reach peak V̇O_2_
TTpeakV̇O_2_: Accumulated time at peak V̇O_2_
UMTT: University of Montréal Track Test
V̇O_2_: Oxygen consumption
V̇O_2max_: Maximum oxygen consumption
vVT_2_: Velocity associated to the second ventilatory threshold
